# Flourish, fight or flight: health in self-employment over time—associations with individual and business resources

**DOI:** 10.1007/s00420-023-02041-z

**Published:** 2024-01-24

**Authors:** Claudia Bernhard-Oettel, Louise Bergman, Constanze Leineweber, Susanna Toivanen

**Affiliations:** 1 Department of Psychology, Stockholm University, Stockholm, Sweden; 2 School of Health, Care and Social Welfare, Mälardalen University, Västerås, Sweden

**Keywords:** Self-employment, Self-rated health, Business success, Longitudinal study, Sweden

## Abstract

**Purpose:**

Using COR theory to study developments of health and other key resources in self-employed workers in Sweden over 6 years, this study: (1) explored whether the heterogenous group of self-employed workers contained subgroups with different health trajectories, (2) investigated whether these were more typical for certain individuals (with respect to age, gender, sector, education, employment status), and (3) compared the different health trajectories regarding resource development in mental well-being, business resources, employment status, work ability.

**Method:**

The study used data from the Swedish longitudinal occupational survey of health (SLOSH) and included participants working as self-employed or combiner (*N* = 2642).

**Result:**

Five trajectories were identified with latent class growth curve model analysis (LCGM). Two health trajectories with (1) very good, respective (2) good stable health (together comprising 78.5% of the participants), (3) one with moderate stable health (14.8%), (4) one with a U-shaped form (1.9%), and (5) one with low, slightly increasing health (4.7%). The first two trajectories flourish: they maintained or increased in all key resources and were more likely to remain self-employed. Trajectories three and five consist of those who fight to maintain or increase their resources. Workers in the U-shaped health trajectory show signs of fight and flight after loss in health and other key resources.

**Conclusions:**

Studying subgroups with different resource developments over time was suitable to understand heterogeneity in self-employed workers. It also helped to identify vulnerable groups that may benefit from interventions to preserve their resources.

## Introduction

In the European Union, approximately 15% of workers are self-employed, meaning they run their own businesses to make a profit (Eurofound 2012). Other developed countries show slightly higher (e.g., New Zealand at 19%, Korea at 23%) or lower (USA and Canada at 7%) self-employment rates (OECD 2022). Encouraging self-employment is common in developed countries as it fuels economic growth and job creation (Binder and Blankenberg 2021), making it an important component of welfare states. In Sweden, around 10.5% of the workers are self-employed (OECD 2022), and most of them work on their own (Ekonomifakta 2023). This means that one in every ten Swedish workers contributes to the welfare system through self-employment, although at the same time, their access to social security systems (e.g. paid leave during sickness, unemployment and pension insurance) is limited (SOU, 2019). With less protection in Sweden and elsewhere (Khan et al. 2021), being healthy and taking care of one’s health is uniquely imperative for the ability to work, run a business and generate an income (Goncalves and Martin 2018), calling for more research on the health of self-employed workers.

To date, however, knowledge about the health status of and health developments in the self-employed is still limited (Torrès and Thurik 2019). Previous studies mostly use self-reported well-being and health data in cross-sectional settings (e.g., Bujacz et. 2020) or with short-term follow-ups of some weeks or months (for a review, see Stephan 2018), thus missing long-term developments. Conversely, studies spanning years or decades primarily use registry data, such as diagnoses, healthcare visits, or mortality data of self-employed workers (e.g., Gauffin and Dunlavy 2021; Toivanen et al. 2016), thus investigating *consequences* of ill-health rather than *health developments* over time. Yet, health developments may relate to entrepreneurial actions such as increasing the turnaround, putting in more work, or quitting long before diagnoses develop, or mortality rates can be studied. Hence, to gain a deeper understanding of how entrepreneurial health links to entrepreneurial experience and actions, we need more research that can monitor general health trends over extended periods, while also tying them to developments in well-being and work circumstances that workers themselves can change (Stepherd and Patzel 2015).

Treating the self-employed as a largely homogenous population is another limitation in previous research. The self-employed are a highly diverse group (Khan et al. 2023) in which health and well-being varies in men and women (Litsardopoulos et al. 2021), may depend on education (Ravina-Ripoll et al. 2021), predictability and control over work load (Stephan 2018), but also business characteristics such as type of business and sector (Gauffin and Dunlavy 2021; Toivanen et al. 2016). To address this heterogeneity, studies utilizing a person-centred methodology (Morin et al. 2018) are needed. Such an approach allows to identify subgroups of self-employed workers with different health trajectories over time. This approach also permits to explore whether different health trajectories lead to or are accompanied by varying developments in other aspects that may relate to the sustainability of self-employment, such as business predictability, personal work abilities or well-being. Taking such an approach would answer the call to study variability and change in the self-employed from a more dynamic perspective (Stephan 2018).

Consequently, our study seeks to uncover and describe trajectories in self-rated health over a six-year period among self-employed workers, and to determine if individuals with specific characteristics (age, gender, education, work status) are more prevalent in certain health trajectories. Based on the Conservation of Resources Theory (COR, Hobfoll 1989), we furthermore explore how resource development in health trajectories corresponds to or sets off resource gains or losses in other domains linked to well-being (mental health, sleep), personal resources (work ability), business resources (order flow variation, income security), and conditional resources related to employment (change in self-employment status).

## Theoretical background

### Conservation of resources theory

The conservation of resources theory (COR, Hobfoll 1989) is an integrated resource theory that views resources as part of a greater dynamic process to build health and well-being. Resources are entities of central value in their own right (e.g. health) or act as means to achieve such a value (e.g. money) (Hobfoll 2002). Threat of resource loss, net loss of resources, or the inability to rebuild resources after a loss are expected to cause stress and since individuals seek for protection and enhancement of the self, they seek to obtain, retain, grow and protect key resources (Hobfoll 1989). Such key resources are central values, and apart from health, these can be objects (e.g., a car), conditions (e.g., employment), personal assets (e.g., skills, abilities) or energies (e.g., time, information, money) that have no intrinsic own value but are used to gain other resources. In addition, an important implication of COR-theory is that resources link to other resources and interact in their development over time to build stable resource reservoirs, net gains or loss spirals (Hobfoll 1989).

**Fig. 1:**
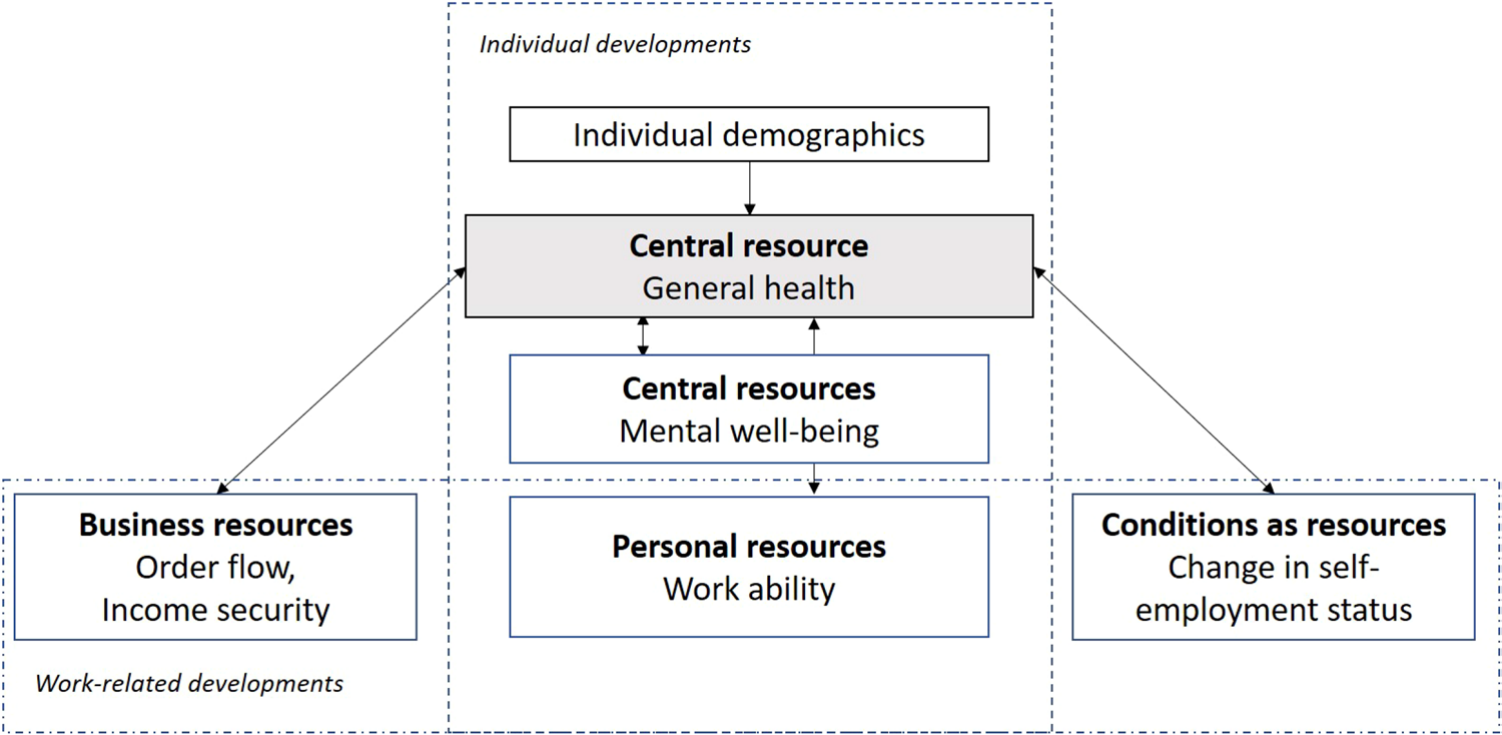
Conceptual model on individual and work-related developments of resources

Mapping this onto the work situation of the self-employed, health is a central resource for the self-employed that can decline, be maintained or strengthened over time (see Fig. 1). Using the person-centred approach, we first investigate whether there are subgroups among self-employed with different health developments, for example, stable health, net gains or losses as suggested in COR-theory. Next, we use individual characteristics that are known to influence overall health differences to validate these health trajectories. To further investigate the existence of stable resource reservoirs, net gains or loss spirals, we study how trajectories in general health relate to developments in other key resources. More specifically, we study co-developments in mental well-being (depressive symptoms, sleep disturbances), which—according to COR-theory—should be closely interlinked with physical health (Hobfoll 1989, 2002). For the work domain, we study how different health trajectories are associated with energy resource developments (in time or money), here referred to as *business resources* (order flow, security of income). We also study links to *conditional resources* related to employment conditions (change in self-employment status). Finally, we investigate *personal resources* (physical and mental work ability) as these are important resources that can change along with developments in individuals and their work situations. In Fig. 1, these abilities therefore are portrayed as both an individual and work-domain resource.

### Central resources: health trajectories over time

To date, there are few studies on health differences and changes in the self-employed. Among the existing studies, many focused on mental health in cross-sectional settings (Stephan 2018), compared health at two-time points only or studied health changes relative to a specific event, such self-employment start or exist (for a recent study, see Nikolova et al. 2021). Evidence on health developments following self-employed workers over extended time periods (e.g. several years) is scarcer and mainly comes from register data. Comparing self-employed and wage workers, mortality and inpatient care rates were found to be higher in the self-employed (Gauffin and Dunlavy 2021). Conversely, other studies reported lower mortality rates in self-employed workers than in wage workers (Goncalves and Martins 2018; Toivanen et al. 2016). It was also found that self-employed workers with limited liability companies had a somewhat lower mortality rate than sole proprietors (Gauffin and Dunlavy 2021), but a less unequivocal picture emerged when sector/industry (Toivanen et al. 2016) and income gradient (Gauffin and Dunlavy 2021) were added to the analyses. In sum, this indicates that heterogeneity in self-employment is worth studying in more detail. However, if the aim is to learn more about health in self-employed workers, these studies have several shortcomings. First, the available register data helps to study proxy measures or consequences of ill-health, which only partly matches the definition of health used by the WHO (WHO, 1948) (being more than the absence of disease). Second, especially measures of mortality can only portray an outcome, that overlooks the importance of health as a resource (Hobfoll 1989), and the agentic role of workers to protect it. Adopting this view, a recent 16-year follow-up study from Australia (Hessels et al. 2020) shows that self-reported general and physical but not mental health were related to higher earnings in self-employed compared to wage workers. However, although self-employed with and without employees were differentiated, Hessels et al. (2020) did not investigate whether health developed differently in subgroups of the self-employed (e.g. health remains stable, fluctuates, increases or decreases over time). Thus, it remains unknown how prevalent potentially different health trajectories in self-employed workers are, and whether investigating such trajectories would help to better understand developments in other important resource domains. This leads to our first research question:

RQ1. Which different health trajectories can be identified in the self-employed, and how prevalent are these?

### Validating identified health trajectories with demographic characteristics

In Sweden, as in many other countries, men are overrepresented in self-employment (OECD 2017). Regarding gender differences in health, it has been found that hospitalization rates were lower for self-employed women than men (Goncalves and Martin 2018), but mental health declined more in female compared to male self-employed workers during the pandemic (Caliendo et al. 2023). However, such health differences between self-employed men and women might be explained by the fact that they work in different sector (Toivanen et al. 2015): Whereas men are most likely to be self-employed in manufacturing, logistics and transportation, and thus, blue-collar work, self-employed women are most likely working in the service sector (OECD 2017).

Regarding education, the average Swedish self-employed has a secondary education (Toivanen et al. 2016) which was the dominant educational level in Sweden in the past (SCB 2022). However, in recent years, those with post-secondary (academic) education and business ideas, and those with low educational levels and few entry paths into the labor market have been most likely to enter self-employment (Simoes et al. 2016). Variations in educational level also mean variations in socio-economic status and thus differences in health (Rostila and Toivanen 2018).

Although the average Swedish self-employed is over 50 years of age, recent investigations show that younger individual in Sweden seem to be as likely to start a new business as older ones (GEM 2022), thus age differences may matter when health developments are studied.

In sum, health is likely to be related to demographic characteristics, but it is unclear to what extent different health trajectories over time may be more prevalent for certain groups of self-employed workers. Due to insufficient previous research, we rather formulate a research question than a hypothesis.

RQ2. Are certain subgroups of self-employed (older workers, females, blue-collar workers, workers with lower education) more likely to be found in a specific health trajectory?

### Relations of identified health trajectories to other resource developments

#### Associations of health trajectories with resource development in well-being

Given that the work environment of self-employed workers is characterized by many challenges related to uncertainty (Mcmullen and Shepherd 2006), high work load (Bernhard-Oettel et al. 2019), long work hours (Binder and Blankenberg 2021) and a blurred work-non-work boundary (Stephan 2018) mental health and good sleep may be resources that are constantly threatened and may have to be rebuilt after periods of net loss.

Many studies on mental well-being in self-employment found that autonomy was positively related to well-being whereas work demands and uncertainty related to the business were associated with elevated levels of burnout, depressive symptoms, fatigue or stress (Stephan 2018). Differences among the self-employed were also noted; those who saw opportunities with their businesses and those who had stabilized their businesses over time reported less distress (Stephan 2018). Longitudinal studies on job and life satisfaction found that longer time and more persistent careers in self-employment were associated with higher levels in satisfaction (Koch et al. 2021; Litsardopoulos et al. 2021).

Few if any studies have used a person-centred approach to explore the existence of different patterns of well-being in the self-employed. One notable exception comes from a cross-sectional study that identified several distinct profiles in well-being among the self-employed (Bujacz et al. 2020), and workers with happy, satisfied, passionate and flourishing patterns were less depressed and anxious and had a more favorable work and income situation. This aligns with COR theory (Hobfoll 1989) suggesting that resourceful individuals are less vulnerable and more likely to build up further resources.

Little research on self-employed workers has been done on sleep, even though sleep may be an important resource to feel well and energetic for a new work day. Sleep quality and thus well-being may vary between individuals and, depending on stressful challenges, even within individuals over time. A recent study showed that on average, the self-employed may sleep more hours than wage workers; but in periods of psychological distress, their sleep quality deteriorates (Wolfe and Patel 2020). How such sleep disturbances develop over time and link to differences in general health in the self-employed is not known.

RQ3. How do health trajectories in self-employed workers differ regarding developments of depressive symptoms and sleep disturbances?

#### Associations of health trajectories with business resource development

Business resources, particularly in terms of money but also time are valuable energy resources to protect or increase the individual resource reservoir (Hobfoll 1989). The availability of these resources may vary in the group of self-employed workers as a function of order flow and possibilities to secure a steady income stream. Running a shop, working in seasonal industries or offering consultancy implies varying contract and order distribution over the year. This means that some self-employed may encounter unpredictable changes in demand (Goncalves and Martin 2018). Some may adopt strategies such as taking on more clients than necessary to secure economic success (Grant and Ferris 2012). Thus, workload and income flows are unevenly distributed, which may lead to stress (Goncalves and Martin 2018), since important resources are at stake. In line with this, subjective well-being has been related to business success and economic well-being in the self-employed (Patel & Wolfe 2019) and as mentioned above, better health was found to be associated with higher earnings (Hessels et al. 2020). However, how potential subgroups of self-employed workers with different health trajectories struggle with or succeed in accumulating business resources, e.g., client/order flow and income security, has not been studied yet. Thus, our research question reads:

RQ4. How do health trajectories in self-employed workers differ regarding developments of business resources, i.e., order flow and income security?

#### Associations of health trajectories with resource development in employment status

Individuals may fully work in self-employment or as combiners, that is, they combine self-employment with wage work (also called hybrid entrepreneurs, see Folta et al. 2010). Since combiners often enter self-employment stepwise or for a test period, they are believed to differ from those solely working as self-employed (Folta et al. 2010). In Sweden, this group has a fair share of entrepreneurial activity. Keeping a paid job in addition to self-employment may add to income security but could also be strenuous and negative for health. Indeed, results show that combiners reported more work-life interference (Hagqvist et al. 2018) and small but statistically significant higher levels of sleep disturbances, exhaustion and depressive symptoms (Bergman et al. 2021) than those solely being self-employed. Remaining self-employed, working as a combiner or taking on wage work instead of self-employment may help individuals maintain, rebuild or stop loss in health resources. A recent study on involuntary and voluntary exit from self-employment found that well-being was more negatively affected than health among those who left self-employment and that effects were stronger for involuntary exit (Nikolova et al. 2021). Thus, developments in health and conditional resources that are tied to the employment status of self-employment, wage work or a combination of both may be related. Still, subgroups with different health trajectories and switches that also include being a combiner have not been studied. Thus, we ask:

RQ5. How do health trajectories in self-employed workers differ regarding changes in employment status over time?

#### Associations of health trajectories with personal resource development

Next, overall health is often discussed as an important resource in self-employment and a prerequisite to be able to run and develop the business (Shepherd and Patzelt 2015; Torrès and Thurik 2019). Since the self-employed are a heterogeneous group and work in various occupations that may entail either more physical (e.g. for carpenters) or mental work (e.g. for HR consultants) the development of both their mental and physical capabilities may be of importance. Such functional capacities have been termed as physical or mental work ability and its most important determinant is health (Ilmarinen et al. 2008). Assumedly, different health trajectories in the self-employed are related to developments in their work ability, but with few studies at hand, it is difficult to hypothesize potential fluctuations or differences in different subgroups. Thus, we formulate the following research question:

RQ6. How do health trajectories in self-employed workers differ regarding developments in physical and mental work ability?

## Method

### Data collection and sample

For this study, data from the Swedish Longitudinal Occupational Survey of Health (SLOSH) were analyzed. This ongoing longitudinal cohort survey on work organization, work environment, and health included at the time of this study a cohort of 40,877 individuals initially representative of the Swedish working population. The SLOSH questionnaire data have been collected bi-annually since 2006, and participants can be employed, self-employed or not actively participating in the workforce (e.g., due to retirement, unemployment or parental leave). Thus, data on health developments can be collected at each wave irrespective of employment status.

Our study population consisted of respondents who indicated in any of the waves 2010–2018 that they were self-employed or combining self-employment with wage work (henceforward termed combiners). We followed respondents from the time point at which they for the first time reported to be self-employed or combiner (T0) and provided a valid account of self-rated health at that time point. We identified 2642 participants who met these selection criteria. Modelling health over time for up to 8 years (five waves) would have required considerable data imputation. Therefore, the follow-up was restricted to 6 years (four waves of data), rendering an average data point coverage of 72.8% of possible data points (see Table 1).

**Table 1:**
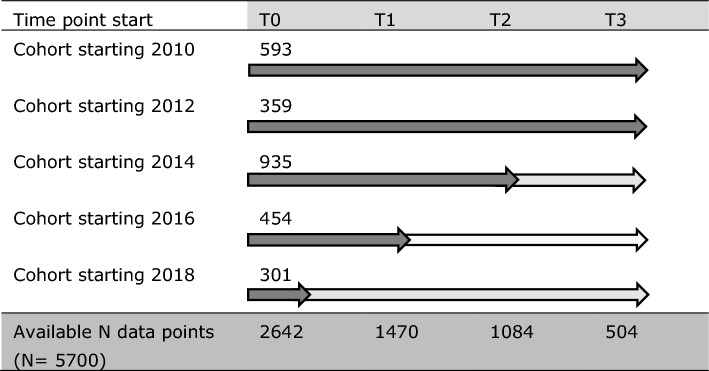
Sample distribution over time and available data points

### Sample characteristics

Descriptive statistics are reported for T0, the time point at which the participants first reported to be self-employed (*N* = 1984, 75.1%) or combiner (*N* = 658, 24.9%). The sample had an average age of 52 years, less than half of the respondents were women (39%) and 55% of all respondents were white-collar workers. Regarding their educational level, 34% had a high school degree (upper secondary education) as their highest education, 31% had a shorter university education (3 years or less), and 35% had a longer university education (more than 3 years). These sample characteristics mirror the fact that self-employed often have lower education than those in wage work, and that women and young individuals are underrepresented in this group in Sweden and the EU (Andersson and Gamerov 2017).

### Measures

*Health resources. Self-rated health* was measured with a single item (“How would you rate your general state of health?”). Answers are given on a five-point scale ranging from ‘very good’ to ‘very bad’. The scale was reversed so that higher values indicate better self-rated health. Various earlier studies have used this item and concluded that it can be considered a reliable and valid global health measure (Idler and Benyamini 1997).

*Demographic variables* were obtained from register data at T0 and treated as time-invariant variables. *Age* was measured in years. *Sex* (0 = male, 1 = female) and *socioeconomic position* (blue-collar (0) versus white-collar (1) workers based on the Swedish socioeconomic classification) were dichotomous variables. *Education* was recoded into a categorical variable (high school education (0), shorter university education of 3 years or less (1) and longer university education of more than 3 years (2)).

*Well-being resources. Depressive symptoms* were measured with six items (e.g. “How much have you been troubled by lethargy or low energy?”) from the Symptom Checklist-Core Depression Scale (Magnusson Hanson et al. 2014). Respondents rated each item on a 5-point Likert scale ranging from (1) “not at all” to (5) “very much”. Means scores were calculated based on these six items and reliabilities were satisfactory at all four measurement points: α_T1_ = 0.91, α_T2_ = 0.90, α_T3_ = 0.89, and α_T4_ = 0.91. *Sleep disturbances* were measured with four items reflecting disturbed sleep during the last 3 months (e.g. difficulties to fall asleep, waking up early) from the Karolinska Sleep questionnaire (Kecklund and Åkerstedt 1992). Respondents rated each item on a 5-point Likert scale ranging from (1) “never” to (6) “always/5 times a week or more”. Means scores were calculated based on these four items and reliabilities were satisfactory at all four measurement points: α_T1_ = 0.85, α_T2_ = 0.85, α_T3_ = 0.84, and α_T4_ = 0.84.

*Business resources* were measured at each time point and treated as time-varying variables. Order flow was operationalized as order variation and need for order. *Order variation* is differentiated between constant order flow over the year (1) and seasonal/varying order flow (2). *Need for order* measured how often the self-employed had no order although they needed one. Answers were given from 1 (very seldom) to 4 (very often). *Income security* was also measured with a single item (“How secure is your income for the coming year?”) and respondents indicated their answer on a scale from 1 (very insecure) to 4 (very secure).

*Conditions as resources*. *Employment status* was a categorical variable to differentiate between self-employed vs combiner at T0. From T1–T3, a third category was added for those who entered wage work.

*Personal resources. Workability* was measured with two items (Ebener and Hasselhorn 2019), one targeting work capacity vis-à-vis physical demands at work (“How would you rate your work capacity concerning physical demands?”) and the other one targeting capacity vis-à-vis mental work demands (“How would you rate your work capacity concerning mental demands?”). Respondents indicated their answer on a five-point scale between “very good” (1) and “very bad” (5). Coding for both variables was reversed so that higher values reflect better work ability.

### Statistical analysis

To answer our first research question (RQ1), a latent class growth curve model analysis (LCGM) was employed. This analysis is increasingly used as a technique for modelling systematic inter- as well as intraindividual differences in change over time (Nagin 2009). It allowed us to find homogeneous latent classes of individuals who differ in their self-rated health trajectories across the four-time points. To find the most appropriate number of latent classes with distinct self-rated health trajectories, we made use of various criteria. First, we inspected ABIC (Adjusted Bayesian Information Criterion), where decreases indicate a better fit of a model to the data. Next, entropy values should preferably be above .90, total count percentages should contain at least 1% of the participants in a trajectory, average posterior probabilities for the trajectories are recommended to be above .70, which indicates that individuals with similar trajectories indeed are grouped together (Jung and Wickrama 2008). We also considered the Lo-Mendell-Rubin adjusted likelihood ratio test (LMR), and the parametric bootstrapped likelihood ratio test (BLRT), in which values > .05, imply that *k* trajectories are enough compared to *k* + 1 trajectories (Jung and Wickrama 2008). Finally, we assessed replicability using the optseed option to ensure that solutions do not represent local maxima.

Next, for answering RQ2, we compared whether individuals in the found health trajectories differed in time-invariant demographic variables (age, gender, blue- collar work, education) at T0 using the BCH procedure (Asparouhov and Muthén 2021). The BCH method avoids shifts in latent classes in the final stage that may occur when using the 3-step method (DU3step and DE3step) for comparing latent classes in levels of auxiliary variables. The BCH method is also advantageous when the variance of the outcome variable differs substantially across classes, which is the case in our data. It is also recommended for binary variables (Asparourhov and Muthén 2021), particularly when the assumption of conditional independence is violated. In the BCH procedure, an overall significant Chi-square value signals mean differences, which are followed up by pairwise comparisons between the different trajectories. For categorical variables with more than two categories, the results of pairwise tests are expressed as OR (95% CI). For example, if group a is compared to group b in a categorical variable with three categories (1, 2, 3) test results show the odds to cross thresholds to category 2 and category 3 in group a and b.

To answer RQ3–6, the health trajectories were compared in time-variant variables. For continuous variables (depressive symptoms, sleep disturbances, need for order, income security, physical and psychological work ability), we first estimated intercept at T0 and growth T0–T3 (i.e., slope over time) in each of these variables by means of latent growth analysis (LGA) and saved values for intercept and slope. In a second step, we then compared whether the health trajectories differed in the starting values (intercept at T0) and the change over time (slope T0–T3) using the BCH procedure as outlined above.

For the time-variant dichotomous variables and categorical variables (order variation, employment status), we compared the health trajectories at each time point to capture differences at T0 and over time. All analyses were performed in Mplus 8.3.

## Results

### RQ1: Identifying health trajectories

Table 2 displays information on fit indices and estimated size of latent class solutions for all models tested. As can be seen, ABIC decreased when more latent classes were added, and was lowest in the model with six latent classes, followed by the model with five latent classes. In both of these models, the percentage of individuals in each latent class ranged from 3 to 48 percent. Whereas the five-class solution showed better entropy (.953) than the six-class solution (.914), the six-class solution had higher posterior probabilities (.90 and above) than the five-class solution (.76 and above). Still, both models pass the criteria of entropy above .90 and posterior classification values exceeding .70. The model with five latent classes had the lowest LMR-LRT and BRLT values of all models. Also, the significance test for LMR-LRT revealed that the model with the five latent classes was significantly better than the model with four classes. In contrast, LMR-LRT of the model with six latent classes was not significant, thus rejecting the model in favor of the previous one with five latent classes. Further tests for seven or more latent classes did not yield better results and led to problems with model identification. Also, class sizes became very small (the extra class that was identified in the model with seven latent classes contained four individuals only) and thus, did not identify any further meaningful subgroups. In sum, considering all fit indices, the model with five latent classes was identified as the best-fitting model.

**Table 2:** Fit indices for self-rated health trajectories testing different numbers of trajectories (latent class growth analysis with the i,s,q model, *N* = 2642)

No. of classes	ABIC	Entropy	% of total counts	Posterior probability	LMR-LRT	BLRT (df)
1	14,601.999	1.00	1.00	1.00	–	–
2	13,242.739	.799	[.19; .81]	[.87; .96]	1335.688***	1378.068(4)***
3	12,653.493	.701	[.13; .58]	[.80; .86]	589.355**	608.055(4)***
4	8214.099	.774	[.05; .52]	[.84; .97]	3951.687	4077.070(4)***
**5**	**5396.784**	**.953**	**[.03; .48]**	**[.76; .99]**	105.058*	108.391(4)***
6	5289.942	.914	[.03; .48]	[.90; .99]	141.705	146.201(4)***

Bold values indicate the chosen solution; ABIC (Akaike Bayesian Information Criterion), LMR-LRT (Lo–Mendell–Rubin likelihood ratio test), BLRT (bootstrapped likelihood ratio test)

***p < .001.**p < .01.*p < .05

Solution with 7 classes resulted in local maxima and included an additional class with *N* = 4 individuals

**Fig. 2:**
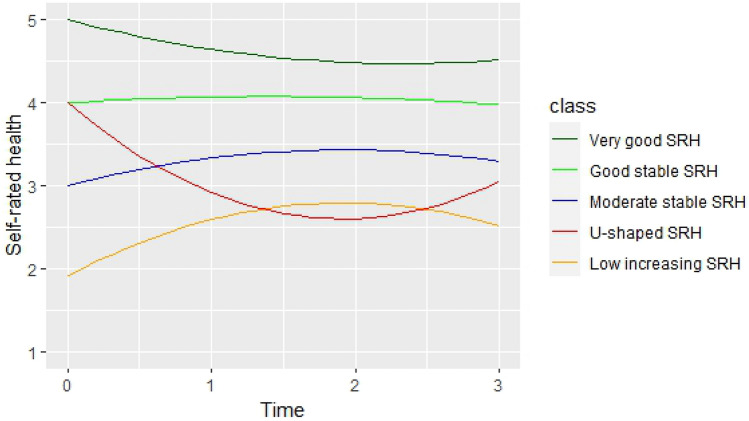
Graphical visualization of the self-rated health trajectories across time (T0–T3)

Figure 2 illustrates the trajectories in self-rated health of the five latent classes. The first class (C1 “Very good slightly decreasing health”) includes 761 individuals (28.8%) who have the highest values in self-rated health at T0, that slightly reduces over time, even though this class still has best self-rated health of all groups at the end of the study period (T3). The second class (C2 “good stable health”) consists of 49.7% of the sample (*N* = 1313) with a stable and good self-rated health trajectory. The third class (C3 “moderate stable health”) incorporates 393 individuals (14.9%) with medium levels of self-rated health that remained rather stable over time. The fourth class (C4 “U-shaped health”) is the smallest with only 50 individuals (1.9%). At the start, these individuals reported good self-rated health that declined at the following two time points and then increased somewhat towards the end (T3). The fifth class (C5 “low increasing health”) consists of 125 individuals (4.7%) with a trajectory of lowest self-rated health over time. Although they reported somewhat improved levels of health at follow-up measurements, their health ratings still are the lowest of all groups at the last time point, T3.

### RQ2: Validating the health trajectories with demographic characteristics

Next, we analyzed the different trajectories of self-rated health with respect to potential differences in demographic variables. The five health trajectories differed significantly in all variables except age (see Table 3).

**Table 3:** Comparison of demographic background (at T0)

	(1) Very good SRH	(2) Good stable SRH	(3) Moderate stable SRH	(4) U-shaped SRH	(5) Low increasing SRH	BCH	Pairwise comparisons
Size *N* (%)	761(28.8)	1313(49.7)	393(14.9)	50(1.9)	125(4.7)		
Age in years	52.4	52.8	53.1	53.1	51.2	3.620, ns	
Sex (% female)	40.6	34.7	29.5	49.5	40.0	17.83**	3 < 1,4,5;2 < 1
Type of work (% white collar)	63.3	57.5	50.3	55.3	49.6	21.67***	1 > 2,3,5;2 > 3
Education at T0						**Manual BCH test**	**OR comparisons for reaching next level**
(1)Up to high school	27.6	33.8	43.8	30	33.6	Having more than high school education	1 > 2 > 3
(2) University < 3 yrs	29.2	28.1	27.2	32	36.8		
(3) University > 3 yrs	43.2	38.1	29.2	38	29.6	Having more than 3yrs university education	1 > 2,3,5

^*^p < .05; **p < .01; ***p < .001

Those with very good, slightly decreasing health were more likely to have a longer university education, had the highest share of white-collar workers and the percentage of women in that group was higher than in the groups of good stable and moderate health. In the group with good stable health, 35% were women, almost two in three worked as white-collar workers and most workers in this group either had a longer university education, or an education up to high school. Thus, their level of education was lower than for those in very good slightly deceasing health but higher than for those in moderate stable health. The share of women in the group with a moderate stable health trajectory was almost 30% and there were 50% white-collar workers in this group. The moderate stable health trajectory had the highest share of workers with a lower education. The U-shaped trajectory included significantly more women than the trajectory with moderate stable health. Among those with a low but increasing health, about 50% worked in blue-collar work (which is comparable to the moderate stable health trajectory) and 40% were women (which equaled the gender distribution of the very good health trajectory). Most individuals in this group had a shorter university education.

### RQ3: Associations of health trajectories with resource development in well-being

To answer RQ3, we investigated whether the trajectories of self-reported health differed in depressive symptoms and sleep disturbances at T0 and in their development over time (see Table 4).

**Table 4:** Comparison of depressive symptoms, sleep disturbances in value at T0 and development over time (slopeT0–T3)

	All	(1) Very good SRH	(2) Good stable SRH	(3) Moderate stable SRH	(4) U-shaped SRH	(5) Low increasing SRH	Chi square (BCH)	Pairwise comparison
Size *N* (%)	2642(100)	761(28.8)	1313(49.7)	393(14.9)	50(1.9)	125(4.7)		
Depressive symptoms, range 1–5 (not at all–very much)								
Value at T0	1.80***	1.51	1.74	2.15	2.40	2.60	549.84***	1 < 2–5; 2 < 3–5;3 < 5
Slope (T0-–T3)	− .03**	− .02	− .03	− .04	− .02	− .05	178.79***	1 = 4; 2 = 4; all else different
Sleep disturbances, range 1–6 (never–always/5 times a week or more)								
Value at T0	2.48***	2.17	2.45	2.81	3.08	3.21	313.96***	3 = 4; 4 = 5; all else different
Slope (T0–T3)	.00	.02	.00	− .01	.03	− .04	55.16***	1 > 2,3,5; 2,3,4 > 5

^***^p < .001; **p < .01; *p < .05

We found that individuals in different health trajectories displayed significant differences in depressive symptoms at the start (χ^2^ = 549.84, *p* < 0.001), and developed differently over time (χ^2^ = 178.79, *p* < 0.001). The trajectories with very good and stable good health displayed significantly lower levels of depressive symptoms at the start than the other groups. Individuals in the moderate stable health trajectory reported significantly lower levels of depressive symptoms than those with low, slightly increasing health. As expressed in the negative significant slope (-0.03, *p* < 0.01), depressive symptoms decreased significantly in all groups over time. However, the decrease was more pronounced for workers with moderate stable health and low but increasing health trajectories. Similarly, sleep disturbances differed at T0 (χ^2^ = 313.96, *p* < 0.001) and developed differently over time (χ^2^ = 55.16, *p* < 0.001) for the five health trajectories. At the start, they were most pronounced in the trajectory of low, slightly increasing health, which differed from the trajectories with very good slightly decreasing as well as good and moderate stable health trajectories. Also, the U-shaped health trajectory was characterized by more sleep disturbances than the trajectories with very good and good health. Over time, sleep disturbances decreased most for those in low but slightly increasing health, which differentiated them from all other groups. In contrast, sleep disturbances increased for the very good slightly decreasing health and the U-shaped health trajectories.

### RQ4: Associations of health trajectories with business resource development

Next, we investigated order flow and income security differences over time in the five health trajectories (see Table 5).

**Table 5:** Comparison of order flow variation and income security at T0 and regarding their development over time (T0–T3, slope)

	All	(1) Very good SRH	(2) Good stable SRH	(3) Moderate stable SRH	(4) U-shaped SRH	(5) Low increasing SRH	Chi square (BCH)	Pairwise comparison
Size *N* (%)	2642 (100)	761 (28.8)	1313 (49.7)	393 (14.9)	50 (1.9)	125 (4.7)		
Order flow Order variation (% seasonal/varying)								
T0	41.22	37.8	42.5	49.3	26.5	50.0	13.43**	1 < 3,5; 2 > 3
T1	40.18	32.9	40.0	44.9	39.1	44.0	8.35n.s	
T2	38.56	27.5	38.8	45.6	39.2	41.7	14.44**	1 < 2,3
T3	46.01	26.0	36.6	57.2	64.0	46.6	15.61**	1,2 < 3
No order although needed, range 1–4 (very seldom–very often)								
Value at T0	1.71***	1.65	1.70	1.79	1.76	1.83	21.84***	1 < 3,5; 2 < 3,5;
Slope (T0–T3)	− .04**	− .03	− .04	− .04	− .03	− .04	6.1n.s	
Income security, range 1–5 (very insecure–very secure)								
Value at T0	2.85***	2.95	2.86	2.74	2.77	2.67	50.35***	1 > 2,3,5; 2 > 3,5
Slope (T0–T3)	.06***	.06	.06	.06	.05	.06	7.23ns	

*p < .05; **p < .01; ***p < .0

As shown in Table 5, variation in order and thus work load differed significantly at all time points but T1 between the groups. More specifically, those in the very good and good health trajectories had significantly lower variation, and thus more steady order distributions over the year than those with moderate and low but increasing health. Likewise, those with very good and good health trajectories less often reported situations in which they had no orders although they needed some. This differentiated them from the workers with moderate and low but increasing health trajectories. The slope was significantly negative, thus, having no orders occurred less often over time and this development did not differ across groups, as the χ^2^ test shows. Income security differed right from start (T0) between the groups (χ^2^ = 50.429, *p* < .001). It was highest for those in very good slightly decreasing health. This differentiated them from all other groups expect for the small group with the U-shaped health trajectory. Income security was also higher for those in good stable health than for the moderate stable and low but increasing health trajectories. The significant and positive slope (.11, *p* < .01) indicated a significant increase in income security over time, and the difference test (χ^2^ = 7.403, *p* > .05) revealed that this increase was comparable in size across all groups.

### RQ5: Associations of health trajectories with resource development in conditions related to employment status

As Table 6 displays, all health trajectories were rather similar at T0: about three out of four individuals were fully self-employed and one out of four combined organizational employment with self-employment. Two years later (T1), employment status differed significantly between those in the U-shaped health trajectory and the ones with a low but increasing health trajectory: The OR for working in self-employment or as combiners was significantly lower (OR 0.32, 95% CI 0.12–0.83, see supplementary material) for those in U-shaped health than for those with low but increasing health. At T2, the five health trajectories did not differ anymore, meaning that the distribution of individuals who were employees, combiners or fully self-employed was again equal across groups. At the last measurement point we found the most pronounced differences between the groups: At that time point, the OR to be working as a combiner was lower for those in very good slightly decreasing health than for workers with moderate stable health (OR 0.44, 95% CI 0.21–0.94) and for workers with low slightly increasing health (OR 0.11, 95% CI 0.02–0.54). In contrast, the OR to be a combiner was higher for those in the moderate stable than in a good stable health trajectory (OR 4.0, 95% CI 1.7–9.5). Likewise, the OR to be working as a combiner was higher in the group with a U-shaped trajectory than among those with good stable health (OR 16.4, 95% CI 2.5–106.7) and low slightly increasing health (OR 11.3, 95% CI 1.3–97.3).

**Table 6:** Comparison of employment status at each point in time (T0-T3)

	(1) Very good SRH	(2) Good stable SRH	(3) Moderate stable SRH	(4) U-shaped SRH	(5) Low increasing SRH	OR SBO/COM vs EMP	OR COM vs SBO
Employment status at T0 in % (total *N* = 2642)							
Only SBO	77.8	74.2%	75.8	68	70.3		
Combiner	22.2	25.8%	24.2	32.0	29.6		
Employment status at T1 in % (total *N* = 1470)							
Only SBO	64.7	66.1	64.3	47.1	68.7		
Combiner	12.8	13.4	13.0	17.3	16.4		
Employee	22.4	20.5	22.7	35.7	14.9	4 > 5	
Employment status at T2 in % (total *N* = 1084)							
Only SBO	63.2	66.7	65.0	47.6	62.8		
Combiner	11.6	9.6	7.8	11.3	7.8		
Employee	25.2	23.7	27.3	41.1	29.4		
Employment status at T3 in % (total *N* = 504)							
Only SBO	64.5	66.1	47.6	17.1	60.1		
Combiner	11.6	7.1	23.1	55.5	9.9		1 < 3;4 > 1,2,5
Employee	23.9	26.9	29.2	27.4	30		
Change over time in % (T0 vs T3)							
Only SBO (T0)							
SBO (T3)	62.6	61.7	47.7	17.3	55		
Combiner (T3)	7.1	6.3	12.3	55.5	10		
Employee (T3)	17.4	18.2	26.2	18.2	15		
Combiner (T0)							
SBO (T3)	1.9	4.4	0	0	5		
Combiner (T3)	4.5	0.8	10.8	0	0		
Employee (T3)	6.5	8.7	3.1	9.1	15		
Chi square	26.1***	38.8***	18.7***	2.93ns	4.90ns		

Next, we analyzed changes in employment status within each trajectory. As signaled by the χ^2^ values (Table 6, lower half), there were significant differences within the groups with very good slightly decreasing health, as well as good and moderate stable health, but not for the U-shaped and low increasing health trajectories. A closer inspection revealed that it was significantly more likely to remain in self-employment and somewhat more likely to continue as combiner in the first three trajectories. In the U-shaped and low health trajectories, self-employed and combiners were as likely to switch to another employment form as they were to stay in the initial employment.

### RQ6: Associations of health trajectories with personal resource development

Finally, Table 7 shows that respondents in different trajectories differed at T0 in how they judged their work ability regarding physical (χ^2^ = 1250.40, *p* < 0.001) and mental work demands (χ^2^ = 591.35, *p* < 0.001). More specifically, those in the very good slightly decreasing health trajectory judged their work abilities best, and this differentiated them from all other groups. Also, those with good stable health reported better work ability than those with moderate stable health, U-shaped health and low, slightly increasing health. Those with U-shaped and low-increasing health trajectory had similar and lowest ratings of their physical and mental work abilities. Over time, physical work ability had a significant and negative slope, meaning that there was a decrease for all workers. However, differences occurred (χ^2^ = 68.930, *p* < 0.001), since those with a U-shaped health trajectory reported more profound declines in physical work ability than those in other trajectories. Work ability to meet mental demands remained more stable over time, but also here, significant differences were noted for the different health trajectories (χ^2^ = 44.56, *p* < .001), and again, the group with U-shaped health reported a significantly higher decline over time than all others.

**Table 7:** Comparison of work ability for physical and psychological demands at T0 and regarding their development over time (T0–T3, slope)

	All	(1) Very good SRH	(2) Good stable SRH	(3) Moderate stable SRH	(4) U-shaped SRH	(5) Low increasing SRH	Chi square (BCH)
Size *N* (%)	2642 (100)	761 (28.8)	1313 (49.7)	393 (14.9)	50 (1.9)	125 (4.7)	
Work ability for physical demands, range 1 (very bad)–5 (very good)							
Value at T0	4.15***	4.54	4.16	3.74	3.61	3.61	1250.40***; 3 = 4; all else different
Slope (T0–T3)	− .022*	− .03	− .02	− .01	− .07	.00	68.930***; 3 = 5; all else different
Work ability for psychological demands, range 1 (very bad–5 (very good)							
Value at T0	4.35***	4.59	4.35	4.11	3.86	3.95	591.35*** 4 = 5; all else different
Slope (T0-–T3)	.00	.00	.01	.01	− .03	.01	44.56***; 1 < 2,3,4; 4 > 2,3,5

*p < .05; **p < .01; ***p < .00

## Summary and discussion

This study used a person-centred approach with the aim to identify distinct subgroups of self-employed workers with similar development in their health resource over a study period of 6 years. We furthermore investigated how these groups can be characterized in terms of individual demographics, and based on COR theory (Hobfoll 1989), we also investigated whether workers in these different health trajectories also differed in other key resource developments over time. We identified five distinct health trajectories among self-employed individuals, who either flourish, fight or flight to protect and gain resources or stop resource loss.

Approximately 78% of the self-employed in this study had either very good, slightly decreasing health (28.8%) or good, stable health (49.7%), which is good news from a public health perspective. Both trajectories were primarily composed of white-collar workers with tertiary education, confirming the positive link between health and human capital in terms of education (Hessel et al. 2020). They had low levels of depressive symptoms and sleep disturbances, which remained stable or even decreased further, reported the best wor ability and their business circumstances were most predictable and favourable over time. In sum, these results illustrate what COR theory predicts, namely that resourceful individuals are less prone to resource loss and more capable of resource gain (Hobfoll 2001). Not surprisingly, over 60% remained solely self-employed throughout the study period and it can be concluded that individuals in these health trajectories were flourishing.

Further, we found two health trajectories (moderate stable and low slightly increasing health) comprising about 20% of the workers in our sample who were fighting in different ways to protect or gain resources. Those with moderate yet stable health trajectory largely reflected the average profile of Swedish self-employed individuals—male, blue-collar workers with secondary education (OECD 2017). Their work ability was significantly lower than those who flourish, but remained at a stable level, and the same was found for well-being. Individuals in the moderate stable health trajectory had more order variability over the entire study period than those who flourish, and less income security, even though these business resources improved with time. This aligns with earlier findings on the relationship between health and business stability and success (Dijkhuizen et al. 2018; Hessel et al. 2020). Roughly 50% remained solely in self-employment, and the highest share (compared to all other trajectories) continued to work as a combiner. We thus find several signals that individuals in this trajectory keep struggling with resource protection (Hobfoll 1989). The trajectory of low, slightly improving health also contained many blue-collar workers. Health and well-being developed in parallel: along with small improvements in health, depressive symptoms and sleep disturbances decreased over time. Compared to all others, individuals in this health trajectory reported the least predictable business resources at T0. Over time order flow became somewhat less variable, and income predictability improved. Thus, this health trajectory reflects the fight for resource gain, which proved difficult as there were fewer resources to be invested (Hobfoll 1989).

Finally, for a small group (1.9%) a U-shaped health trajectory was identified, showing significant decreases followed by some recovery between T2 and T3. This trajectory was unique as developments in health, well-being and personal ability did not align as they did in all other trajectories. At T0, health levels were similar to those found in good and very good health trajectories, but depressiveness and sleep disturbances were comparable to the levels found in the low slightly increasing health trajectory. Over time, rather high levels of sleep disturbances increased further while depression only reduced slightly. Likewise, work ability, particularly with respect to psychological work demands, was lowest already at T0 and further decreased with time. The results thus indicate a profound resource loss (Hobfoll 1989) and this most likely relates to the sharp health decline observed later on. In terms of business resources, order flow variations increased over time, which may signal changing business strategy and thus entrepreneurial action such (Shepherd and Pratzelt 2015). Notably, 35% left self-employment between T0 and T1, and at the final timepoint, only 17% in the U-shaped health trajectory remained entirely self-employed. Thus, health declined already before exit (for similar findings, see Nikolova et al. 2021). Altogether, an overall picture emerges that individuals in this health trajectory fight with resource loss (Hobfoll 1989) and choose flight as a strategy to stop further resource depletion.

Overall, these results allow several important conclusions. First of all, a large majority seemed to flourish, largely fitting the description of the healthy self-employed worker with a successful business, sometimes referred to as the ‘heroic’ entrepreneur (Torres and Thurik 2019). Still it has to be noted that 21.5%—more than one in five self-employed workers struggled in varying ways. Our findings contradict an earlier cross-sectional study by Bujacz et al. (2020) on European data, in which a profile that ‘flourished’ only comprised 10% of all self-employed workers, and the prevalence of unhappy or languishing individuals was higher. However, prevalence rates may not be easy to compare since there are important differences in both studies in terms of well-being measures, nature of the data and labour market context.

Interestingly, we found that the gradient in resource loss was comparably steeper than the gradient in net gain. This fits well with the premises of COR theory stressing that resource loss is more significant whereas gains are long-term and rather slow transformations (Hobfoll 2001), perhaps also because they require constant resource investments (Hobfoll 1989). Also, our finding may illustrate that being in poorer health and running a more volatile business is a constant struggle which may mean that not everybody is well-equipped to be self-employed (see also Bujacz et al. 2020 for a similar conclusion). Interestingly, those with steep declines in health were more prone to leave self-employment whereas those with small but constant gains over time persisted to a higher degree. Reasons for this are highly speculative, one may be that the individuals in the U-shaped trajectory had higher education and thus, perhaps better chances to enter wage work. Other reasons may be that those with low but slightly increasing health infact see some net gains. Based on COR theory (Hobfoll 1989) it may also be that they believe that their resources are better protected and developed in self-employment than wage work.

### Strengths and limitations

A strength of this study is its rather big sample of self-employed workers and due to its longitudinal nature, the possibility to study developments over a time frame of 6 years. Also, this is one of the few studies where health developments could be followed even if individuals went back into regular employment. Thus, health trajectories are not only calculated for those who remain self-employed, which otherwise renders a “survival of the fittest” effect, (Goncalves and Martins 2018). Another strength of the study is that it made use of health measures on scales that have been tested extensively and found to be valid even in self-employment, including combiners (Bergman et al. 2021). Also, elaborated statistical analysis methods were used to study intra-individual developments of time so that the stability of developments at different levels of health could be made visible.

However, as with all research, there are a number of shortcomings necessary to discuss for the correct interpretation of our findings. First, there was no information about the starts of the businesses, and T0 in this study is the first time point that an individual in the SLOSH database provided information as self-employed or combiner. Thus, this is a study of self-employment over time, at any time in the career of the self-employed worker. This limit what conclusions we can draw but allows the simultaneous study of self-employed workers at different career stages. Thus, the found trajectories provide a glimpse into health and resource development in self-employment at any given time, which helps to further understand the sustainability of self-employment in this heterogeneous group.

Importantly, the study cannot establish causality but has a rather observational and exploratory character. Although this fits the test for dynamic and parallel inter-developments of resource maintenance gains or losses as proposed in COR theory (Hobfoll 1989), it comes with the disadvantage that it is difficult to pinpoint which resources should be considered as cause vs consequence. However, as described by COR, resources can co-travel and affect each other in a feedback loop, thus, the establishment of causality was not a focus of this study. Although this study has tested differences between the health trajectories with regard to other resources in both levels and developments (slope, change of category), all relationships were modelled within the same points in time, which hampers the detection of delays in parallel resource developments. The U-shaped trajectories hint at such a scenario: Well-being and work ability declines were visible earlier than health declines. Furthermore, two of the identified trajectories were found in rather small groups and larger within-group variances in groups with lower N made the detection of statistically significant group differences more difficult. Another caveat also is that the SLOSH data contains few self-employed workers born outside of Sweden so that the results do not mirror the situation of immigrants. It should further be noted that data reported here was collected before the COVID pandemic so there is no information on how a disruption like the pandemic would affect the health developments under study.

### Theoretical and practical implications

Despite its shortcomings, the study offers valuable longitudinal evidence in the field of self-employment and health, surpassing the timeframe of most prior studies (see also Stephan 2018) and is one of the first to also include work ability, although remaining in business may hinge on that ability (Torres and Thurik 2019).

As can be seen in our study, using a person-centred approach has the merits of providing a more holistic perspective on typical developments (see also Morin et al. 2018), and this approach has hereto been underused for studying the diversity of the self-employed (Khan et al. 2023).

We explored how developments in health correspond to changes in other vital resources, offering a dynamic view of resource maintenance, accumulation, or depletion. Such dynamic approaches have been called for (Stephan 2018) and despite much theoretical debate (Torres and Thurik 2019), the complex interdependence that unfolds between individual health and well-being and economic as well as business developments has not been captured well in earlier empirical studies. Our study can give valuable first insights into this complexity. Potentially also, our study adds to knowledge concerning the interdependence of entrepreneurial health and action (Shepherd and Patzelt 2016) given that we found some associations between poor resource development and exit from self-employment or work as a combiner. However, more research is needed that study health and well-being and delve into the different actions that the self-employed take to protect their health and well-being. Related to that, an important question of our study is what it takes to be a healthy and resourceful self-employed, and to what extent the found differences in good, moderate and low health do exist from the start, perhaps due to selection effects (Torres and Thurik 2019) and persist over time.

We also believe that the theoretical foundations laid out in COR theory and other resource frameworks (Hobfoll 2002) have much to offer for future research studies on health and resource development in self-employed workers. For example, COR theory can be a useful framework for studying various resource types in work and non-work domains that self-employed workers may need to juggle the challenges inherent in running a business (Grant and Ferries 2012).

Another area of development lays in the operationalization of resources and the modelling of their co-dependence. Future studies are needed that allow us to model the development of health and other resources in more detail and with shorter time gaps, for example in multigroup analyses or with parallel growth curves and with tests for delayed and reciprocal relationships. Furthermore, additional resources may need further investigation since the operationalizations chosen in this paper are by no means exhaustive (for an overview of other potential resources, see Hobfoll 2001).

For policymakers and practitioners, our results also offer important insights. While the majority flourish, it is crucial to note that many of them were white-collar workers with higher education and predictable business resources concerning time allocation and income security. Those who fight for resource protection and gain were more likely to be blue-collar workers, and those with less predictability in order flow and income. Based on our results, depressive symptoms, sleep problems and diminishing work ability may be early signs of fighting despite similar health ratings, order and income security as in flourishing groups. In fact, also Nikolova et al. (2021) observed declines in well-being prior to exit from self-employment, and the step to finally leave is not taken light-heartedly since it may be considered a personal failure. Here, perhaps early interventions may be needed that target the health and well-being of self-employed workers (Williamsson et al. 2021) or as in the case of the ones in the low health trajectory, help to stabilize or increase health or build business resources. An interesting avenue for practitioners and researchers alike may be to test whether addressing well-being, bolstering business resources, or both, could positively impact resource development. This may also further help to disentangle whether business resources and actions or entrepreneurial health are the primary resource to spark other resource gains.

Finally, as has been noted before, self-employed workers’ health developments also have implications for public health policies and social security regulations (Goncalves and Martins 2018; Khan et al. 2023). Sweden is a country with a rather generous well-fare system even though it is more tailored to the conditions and needs of those in wage work. However, the high prevalence of good and very good health trajectories in this study may be a function of the contextual background. Yet, even in Sweden, we could identify a share of self-employed workers who fight, and those with remarkable health decline use flight as a way of stopping losses. Albeit this was a small number, in the long run it may mean that individuals leave when they encounter health difficulties, which would imply that innovative potential in the society gets lost. Across Europe and the entire world, the percentages of workers in self-employment are higher in countries with less advanced security regulations (OECD 2022), and this calls for more research in different labor market settings with a person-centred approach to understand how prevalent low or declining health trajectories are. Our results also call for more research to understand causes of negative developments and to test suitable interventions so that self-employed workers’ health and resourcefulness is protected.

## Data Availability

Given restrictions from the ethical review board and considering that sensitive personal data are handled, it is not possible to make the data freely available. Access to the data may be provided to other researchers in line with Swedish law and after consultation with the Stockholm University legal department. Requests for data, stored at the Stress Research Institute, Department of Psychology, should be sent to registrator@su.se with reference to this publication or directly to the corresponding author.

## References

[CR1] Andersson M, Gamerov L (2017) Färre kvinnor än män driver företag. [Fewer women than men run a business]. https://www.scb.se/hitta-statistik/artiklar/2017/Farre-kvinnor-an-man-driver-foretag/

[CR2] Asparouhov T, Muthén B (2021) Auxiliary variables in mixture modeling: using the BCH method in mplus to estimate a distal outcome model and an arbitrary secondary model.mplus Web Notes: No. 21 Version 11.

[CR3] Bergman LE, Bernhard-Oettel C, Bujacz A, Leineweber C, Toivanen S (2021). Comparing depressive symptoms, emotional exhaustion, and sleep disturbances in self-employed and employed workers: application of approximate Bayesian measurement invariance. Front Psychol.

[CR4] Bernhard-Oettel C, Leineweber C, Westerlund H (2019). Staying in or switching between permanent, temporary and self-employment during 2008–2010: associations with changing job characteristics and emotional exhaustion. Econ Ind Democr.

[CR5] Binder M, Blankenberg A-K (2021) A service of zbw Self-employment and subjective well-being standard-Nutzungsbedingungen: self-employment and subjective well-being $. http://hdl.handle.net/10419/228453

[CR6] Bujacz A, Eib C, Toivanen S (2020). Not all are equal: a latent profile analysis of well-being among the self-employed. J Happiness Stud.

[CR100] Caliendo M, Graeber D, Kritikos AS, Seebauer J (2023). Pandemic depression: COVID-19 and the mentalhealth of the self-employed. Entrepreneurship theory and practice.

[CR7] Dijkhuizen J, Gorgievski MJ, van Veldhoven M, Schalk R (2018). Well-Being, personal success and business performance among entrepreneurs: a two-wave study. J Happiness Stud.

[CR8] Ebener M, Hasselhorn HM (2019). Validation of short measures of work ability for research and employee surveys. Int J in Environ Res Public Health.

[CR9] Ekonomifakta (2023) https://www.ekonomifakta.se/fakta/foretagande/naringslivet/foretagare/. Accessed 8 Nov 2023

[CR10] Eurofond (2012) The European industrial relations dictionary. www.eurofond.europa.eu

[CR11] Folta TB, Delmar F, Wennberg K (2010). Hybrid entrepreneurship. Manag Sci.

[CR12] Gauffin K, Dunlavy A (2021). Health inequalities in the diverse world of self-employment: a Swedish national cohort study. Int J Environ Res Public Health.

[CR13] GEM (2022) Global Entrepreneurship Monitor 2021/2022 Global Report: Opportunity Amid Disruption.

[CR14] Goncalves J, Martins P (2018) The effect of self-employment on health: evidence from longitudinal social security data. In. IZA DP No. 11305. Bonn, Germany: IZA – Institute of Labor Economics.

[CR15] Grant S, Ferris K (2012). Identifying sources of occupational stress in entrepreneurs for measurement. Int J Entrepreneurial Ventur.

[CR16] Hagqvist E, Toivanen S, Bernhard-Oettel C (2018). Balancing work and life when self-employed: the role of business characteristics, time demands, and gender contexts. Social Sciences.

[CR17] Hessels J, Rietveld CA, van der Zwan P (2020). The relation between health and earnings in self-employment. Front Psychol.

[CR18] Hobfoll SE (1989). Conservation of resources. A new attempt at conceptualizing stress. Am Psychol.

[CR19] Hobfoll S (2001). The influence of culture, community, and the nested-self in the stress process: advancing conservation of resources theory. Appl Psychol.

[CR20] Hobfoll S (2002). Social and psychological resources and adaptation. Rev Gen Psychol.

[CR21] Idler EL, Benyamini Y (1997). Self-rated health and mortality: a review of twenty-seven community studies. J Health Soc Behav.

[CR22] Ilmarinen J, Gould R, Järvikoski A, Järvisalo J (2008). Diversity of work ability. In Gould R, Ilmarinen J, Järvisalo J, Koskinen S (Eds.), Dimensions of work ability (pp. 13–24). Finnish Centre for Pensions (ETK), The SII (Kela), KTL, FIOH.

[CR23] Jung T, Wickrama KAS (2008). An introduction to latent class growth analysis and growth mixture modeling. Soc Pers Psychol Compass.

[CR24] Kecklund G, Åkerstedt T (1992). The psychometric properties of the Karolinska Sleep Questionnaire. J Sleep Res.

[CR25] Khan TH, MacEachen E, Hopwood P, Goyal J (2021). Self-employment, work and health: a critical narrative review. Work.

[CR26] Khan TH, MacEachen E, Premji S, Neiterman E (2023). Self-employment, illness, and the social security system: a qualitative study of the experiences of solo self-employed workers in Ontario, Canada. BMC Public Health.

[CR27] Koch M, Park S, Zahra SA (2021). Career patterns in self-employment and career success. J Bus Ventur.

[CR28] Litsardopoulos N, Saridakis G, Hand C (2021). Does the accumulation of self-employment experience impact life satisfaction?. J Bus Ventur Insights.

[CR29] Magnusson Hanson LL, Westerlund H, Leineweber C, Rugulies R, Osika W, Theorell T, Bech P (2014). The Symptom Checklist-core depression (SCL-CD6) scale: psychometric properties of a brief six item scale for the assessment of depression. Scand J Public Health.

[CR30] Mcmullen JS, Shepherd DA (2006) Entrepreneurial action and the role of uncertainty in the theory of the entrepreneur.The Academy of Management Review, 31(1):132–152.

[CR31] Morin AJS, Bujacz A, Gagné M (2018). Person-centred methodologies in the organizational sciences: introduction to the feature topic. Organ Res Methods.

[CR32] Nagin D (2009). Group-based modeling of development.

[CR33] Nikolova M, Nikolaev B, Popova O (2021). The perceived well-being and health costs of exiting self-employment. Small Bus Econ.

[CR34] OECD (2017) Entrepreneurship at a Glance 2017.

[CR35] OECD (2022) Self-employment rate (indicator). 10.1787/fb58715e-en.

[CR36] Patel PC, Wolfe MT (2019). Money might not make you happy, but can happiness make you money. The value of leveraging subjective well-being to enhance financial well-being in self-employment. J Bus Ventur Insights.

[CR37] Ravina-Ripoll R, Foncubierta-Rodríguez MJ, Ahumada-Tello E, Tobar-Pesantez LB (2021). Does entrepreneurship make you happier? A comparative analysis between entrepreneurs and wage earners. Sustainability (switzerland).

[CR38] Rostila M, Toivanen S (2018) Den orättvisa hälsan – om socioekonomiska skillnader i hälsa och livslängd [Unfair health - about socioeconomic differences in health and longevity]. Stockholm: Liber.

[CR39] SCB (2022) Utbildningsnivån i Sverige [Educational level in Sweden].

[CR40] Shepherd DA, Patzelt H (2015). The “heart” of entrepreneurship: the impact of entrepreneurial action on health and health on entrepreneurial action. J Bus Ventur Insights.

[CR41] Simoes N, Crespo N, Moreira SB (2016). Individual determinants of self-employment entry: what do we really know?. J Econ Surv.

[CR42] Statens Offentliga Utredningar SOU (2019). Företagare i de sociala trygghetssystemen, SOU 2019:41. Näringsdepartementet. Stockholm, Elanders Sverige AB. SOU 2019:41.

[CR43] Stephan U (2018). Entrepreneurs’ mental health and well-being: a review and research agenda. Acad Manag Perspect.

[CR44] Toivanen S, Mellner C, Vinberg S (2015). Self-employed persons in sweden-mortality differentials by industrial sector and enterprise legal form: a five-year follow-up study. J Ind Med.

[CR45] Toivanen S, Griep RH, Mellner C, Vinberg S, Eloranta S (2016). Mortality differences between self-employed and paid employees: a 5-year follow-up study of the working population in Sweden. Occup Environ Med.

[CR46] Torrès O, Thurik R (2019). Small business owners and health. Small Bus Econ.

[CR47] Williamsson A, Gish JJ, Stephan U (2021). Let’s focus on solutions to entrepreneurial ill- being! Recovery interventions to enhance entrepreneurial well- being. Entrepreneurship Theory Pract.

[CR48] Wolfe MT, Patel PC (2020). I will sleep when I am dead? Sleep and self-employment. Small Bus Econ.

